# Biomechanical control of paretic lower limb during imposed weight transfer in individuals post-stroke

**DOI:** 10.1186/s12984-020-00768-1

**Published:** 2020-10-27

**Authors:** Hao-Yuan Hsiao, Vicki L. Gray, James Borrelli, Mark W. Rogers

**Affiliations:** 1 Department of Kinesiology and Health Education, University of Texas at Austin, Austin, TX USA; 2 Department of Physical Therapy and Rehabilitation Science, University of Maryland School of Medicine, Baltimore, MD USA

**Keywords:** Stroke, Balance, Weight transfer, Weight bearing, Limb loading, Locomotion, Perturbation

## Abstract

**Background:**

Stroke is a leading cause of disability with associated hemiparesis resulting in difficulty bearing and transferring weight on to the paretic limb. Difficulties in weight bearing and weight transfer may result in impaired mobility and balance, increased fall risk, and decreased community engagement. Despite considerable efforts aimed at improving weight transfer after stroke, impairments in its neuromotor and biomechanical control remain poorly understood. In the present study, a novel experimental paradigm was used to characterize differences in weight transfer biomechanics in individuals with chronic stroke versus able-bodied controls

**Methods:**

Fifteen participants with stroke and fifteen age-matched able-bodied controls participated in the study. Participants stood with one foot on each of two custom built platforms. One of the platforms dropped 4.3 cm vertically to induce lateral weight transfer and weight bearing. Trials involving a drop of the platform beneath the paretic lower extremity (non-dominant limb for control) were included in the analyses. Paretic lower extremity joint kinematics, vertical ground reaction forces, and center of pressure velocity were measured. All participants completed the clinical Step Test and Four-Square Step Test.

**Results:**

Reduced paretic ankle, knee, and hip joint angular displacement and velocity, delayed ankle and knee inter-joint timing, increased downward displacement of center of mass, and increased center of pressure (COP) velocity stabilization time were exhibited in the stroke group compared to the control group. In addition, paretic COP velocity stabilization time during induced weight transfer predicted Four-Square Step Test scores in individuals post-stroke.

**Conclusions:**

The induced weight transfer approach identified stroke-related abnormalities in the control of weight transfer towards the paretic limb side compared to controls. Decreased joint flexion of the paretic ankle and knee, altered inter-joint timing, and increased COP stabilization times may reflect difficulties in neuromuscular control during weight transfer following stroke. Future work will investigate the potential of improving functional weight transfer through induced weight transfer training exercise.

## Background

Stroke is a leading cause of death and serious long-term disability in the United States [1, 2]. Individuals with hemiparesis due to stroke commonly demonstrate difficulty bearing weight on the paretic lower extremity and transferring weight from one leg to the other [3–5]. Reduced paretic limb weight bearing has been associated with functional deficits when rising from a chair [6], standing [7], and walking [8, 9]. The ability to transfer bodyweight between the lower limbs is related to impaired standing and stepping balance [10, 11] and gait performance [3, 12]. In particular, diminished weight transfer to the paretic limb contributes to gait asymmetries, which commonly lead to greater energy expenditure [13]. Previously we reported the ability to transfer weight laterally to the paretic leg during single stance was associated with self-selected walking speed and the capacity to increase walking speed [14]. This may indicate that weight transfer deficits negatively affect forward progression. Indeed, forceful weight shift towards the paretic limb enhanced paretic lower extremity kinetics and muscle activities that contribute to forward progression [15]. Moreover, deficits in paretic limb weight-bearing contribute to lateral and vertical balance instability and are associated with risk of falling in individuals with chronic stroke [16]. These functional limitations can affect community participation and quality of life. Consequently, restoring the capacity to load the paretic limb is an important goal for rehabilitation post-stroke [17–19].

Despite considerable rehabilitation efforts aimed at improving weight transfer following a stroke [10, 20], the impairments in neuromotor and biomechanical control underlying weight transfer dysfunction remain poorly understood. Functional weight transfer requires the coordination of multi-joint actions to absorb the impact force and provide support to the body. In particular, the ankle and knee joints are key contributors to shock absorption [21–24] and body weight support [25]. Increased stiffness in the paretic limb knee and ankle joints has been reported in persons with stroke [26, 27]. Inadequate lower limb joint flexion may disrupt impact force regulation during weight acceptance and lead to instability that ultimately delays and prolongs weight transfer timing during locomotion. Alternatively, excessive ankle and knee joint flexion during loading may precipitate limb collapse and destabilize balance during weight transfer. Thus, both insufficient and excessive joint movement could affect weight transfer processes. In addition to the amplitude of paretic ankle and knee joint angular displacements, abnormalities in the relative timing of these joint motions (i.e., inter-joint coordination) may also contribute to impaired weight transfer following stroke.

Another key factor affecting functional weight transfer is the ability to regulate the center of pressure (COP) beneath the feet in relation to the body center of mass (COM). During locomotion, effective neuromotor control of the lower extremities contributes to regulating COM position and movement relative to the base of support to maintain stability and prevent falling. Compared with able-bodied adults, persons with chronic stroke have a reduced capacity to rapidly shift their COP to the stance limb during gait initiation [28], reflecting abnormalities in balance control during weight transfer. Because hip and ankle musculature regulates COM and COP movements [29], difficulties in controlling hip kinematics and hip-ankle joint coordination may contribute to delayed and reduced weight transfer after a stroke.

To further address the foregoing issues, this study examined the potential biomechanical factors that could affect lower paretic limb weight bearing and weight transfer performance following stroke. After stroke individuals often limit their use of the paretic limb by favoring the use of the less affected leg during stance and gait [30]. An approach that forces individuals to fully load the paretic limb is warranted to reveal the performance capacity and assess the control of weight bearing and weight transfer. Accordingly, we designed a novel system that vertically displaces the support surface underneath one leg and therefore imposes weight transfer. By unilaterally introducing a perturbation that drops the standing support surface, this approach forces a rapid alteration in inter-limb weight bearing distribution and challenges medial–lateral balance control.

The primary purpose of this study was to characterize the kinematics and kinetics of the paretic lower extremity during an externally induced weight transfer towards the paretic limb in chronic stroke compared to age-matched controls. We hypothesized that, compared with able-bodied individuals, those with chronic stroke would show reduced and uncoordinated paretic limb joint angular displacements, and prolonged stabilization time of the COP velocity following an externally induced weight transfer. In addition, relationships between measurements during imposed weight transfer, motor recovery assessment (i.e. Chedoke McMaster Stroke Assessment leg and foot subscale), and clinical limb loading and balance performance (i.e. Four-Square Step Test (FSST) and Step Test (ST)) were explored. We expected that COP velocity stabilization time and CMSA scores would be associated with FSST and ST scores.

## Methods

### Participants

Fifteen individuals with chronic stroke (67.7 ± 7.22 years; 5 females; time since stroke 12 ± 12 years; 4 right paretic; Chedoke McMaster Stroke Assessment score of the leg 4.9 ± 1.3 and foot 3.7 ± 1.8) and fifteen age-matched able-bodied controls (67.7 ± 5.87 years; 4 females; 13 right dominant) participated in the study (Table 1). The inclusion criteria were: (1) Hemiparesis as a result of a stroke greater than 6 months prior to the study for participants with stroke; (2) Able to walk 10 m with or without a walking aid; and (3) Able to stand unsupported for 5 min. Controls were included if they had no self-reported history of a neurological injury or condition. The exclusion criteria were: (1) unable to follow instructions; (2) medical conditions beyond the effects of the stroke precluding participation in regular exercise; and (3) pregnancy. The study was approved by the Institutional Review Board of the University of Maryland Baltimore and all participants provided written informed consent to participate.

**Table 1:** Participants’ characteristics

Stroke	Age	Time since stroke onset (years)	Sex	Paretic Side	CMSA leg	CMSA foot	Control	Age	Sex	Non-dominant side
S00	73	16.8	F	R	5	3	C01	73	M	L
S01	72	3.5	F	R	3	2	C02	67	M	R
S02	58	1.3	M	R	7	5	C03	73	M	L
S03	72	5.1	M	R	6	6	C04	73	M	L
S04	74	9.2	M	L	5	5	C05	55	M	L
S05	68	8.2	M	L	6	4	C06	56	F	L
S06	62	10.2	F	L	6	5	C07	63	M	L
S07	63	2.0	M	L	5	5	C08	64	F	L
S08	71	12.4	M	L	3	1	C09	68	M	L
S09	55	1.8	M	L	5	2	C10	71	M	L
S10	76	51.6	F	L	3	3	C11	69	M	L
S11	59	14.5	M	L	6	7	C12	71	F	L
S12	68	7.7	M	L	3	2	C13	71	M	R
S13	80	21.3	M	L	5	3	C14	73	M	L
S14	65	8.8	F	L	6	2	C15	69	F	L
Mean	67.7	12			4.9	3.7		67.7		
STD	7.2	12			1.3	1.8		5.9		

*CMSA* Chedoke McMaster Stroke Assessment

### Testing procedure

#### Clinical assessment

All participants completed the clinical Step Test (ST) and Four-Square Step Test (FSST) to assess balance and mobility. A transfer belt was worn by all participants performing the clinical tests to ensure their safety. During the ST, participants stood 5 cm from a 7.5-cm-high step, placed one foot onto the step and then returned it to the floor. Participants repeated this movement as fast as possible for 15 s [31]. The number of steps completed in a 15-s period was recorded. Participants performed two trials stepping on and off of the step with each limb. The trial with the greatest number of steps from each limb was used for analysis. The ST has been previously shown to have excellent test–retest reliability [31] and is responsive to change during rehabilitation in people post-stroke [32]. In addition, among several clinical measures, the ST has been found to have moderately strong association with peak vertical ground reaction forces measured from force platforms beneath the paretic limb (r^2^ = 0.76) [11].

During the FSST, participants completed a rapid sequential stepping task clockwise and counterclockwise while avoiding four canes arranged in a cross pattern on the floor with the tips of the canes facing together. The test procedure was demonstrated, and one practice trial was performed prior to administering the test. Two trials were performed, and the best time was taken as the score. The FSST involves the time-dependent capacity to limb loading while stepping in multiple directions and has been shown to be an effective and valid tool for measuring dynamic balance [33].

The Chedoke McMaster Stroke Assessment Impairment Inventory (CMSA) leg and foot subscales were used to assess motor recovery. The CMSA score assesses the range of motion, ability to move in and out of synergistic patterns, and capacity to generate rapid movements. The score ranges from 1 to 7, with 7 representing full recovery and the ability to perform rapid and complex joint movements.

#### Imposed weight transfer assessment

**Fig. 1:**
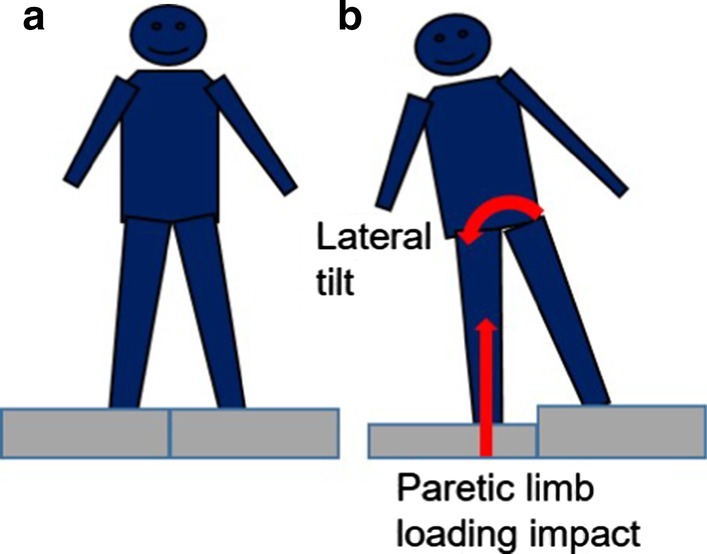
Unilateral platform perturbation system (**a**) induced lateral tilt of the body and forced paretic limb vertical weight-bearing (**b**)

All participants wore an un-instrumented safety harness during the imposed weight transfer assessment. Two movable platforms (height ~ 37 cm) were placed adjacent to each other (see Fig. 1a). Participants stood with one foot on each platform. Each of the standing platforms was held securely to the support structure using ten electromagnets (12 V DC, Magnetech Corp). Disengagement of the magnets, via computer control, released the support surface causing it to drop 4.3 cm vertically. A layer of carpet was glued on the bottom of the drop surface and a foam pad (thickness ~ 5 mm) was placed between the platform and the force plate to reduce the impact sound to less than 70 dB, below the threshold known to elicit a startle response [34, 35]. Following the perturbation, participants typically extended the perturbed limb (paretic or non-dominate for controls) and flexed the non-perturbed limb. During the aerial phase of platform falling, the perturbed foot moved downwards with the platform without ground support. Joint flexion in the perturbed limb (see Fig. 1) and trunk tilt were typically observed following initial ground contact. Participants were instructed to stand naturally with their weight evenly distributed on each leg. An investigator monitored the vertical ground reaction forces that were depicted on the screen to ensure an approximate symmetrical weight-bearing at the start of each trial. Two familiarization trials (one trial for each side) were provided for each participant. Next, four unilateral support surface lowering perturbation trials were delivered to each leg. The order of the drop was randomized and the perturbation was delivered at an unexpected timing. Participants were told to stand in a comfortable position and respond naturally to the drop to maintain their upright posture. An investigator stood in close proximity to the participant to assist as needed. The outline of each participant’s feet was traced to ensure the same initial position. Shoes were worn during the testing protocol, and individuals with ankle–foot orthoses (AFO) were allowed to keep the AFO on during the clinical tests if they felt it was necessary.

### Data recording

#### Kinematic recordings

Body segment position data were recorded using a 10 camera motion capture system (Vicon-USA, Denver, CO). Reflective markers were placed on the forehead and bilaterally on the acromion process, lateral epicondyle of the humerus, distal end of the radius, anterior superior iliac spine, posterior superior iliac spine, greater trochanter (hip), lateral epicondyle of the femur (knee), lateral malleolus (ankle), second metatarsal, and heel. Signals were sampled at 120 Hz for 5 s (including 1–2 s prior to perturbation onset) and then low-pass filtered offline at 6 Hz.

#### Kinetic recordings

Ground reaction forces and center of pressure (COP) data were measured using two AMTI force platforms (Advanced Mechanical Technology Inc., Watertown, MA) located beneath the standing platforms. Signals were sampled at 600 Hz for 5 s (including 1–2 s prior to perturbation onset) and then low-pass filtered offline at 30 Hz.

### Data analyses

**Fig. 2:**
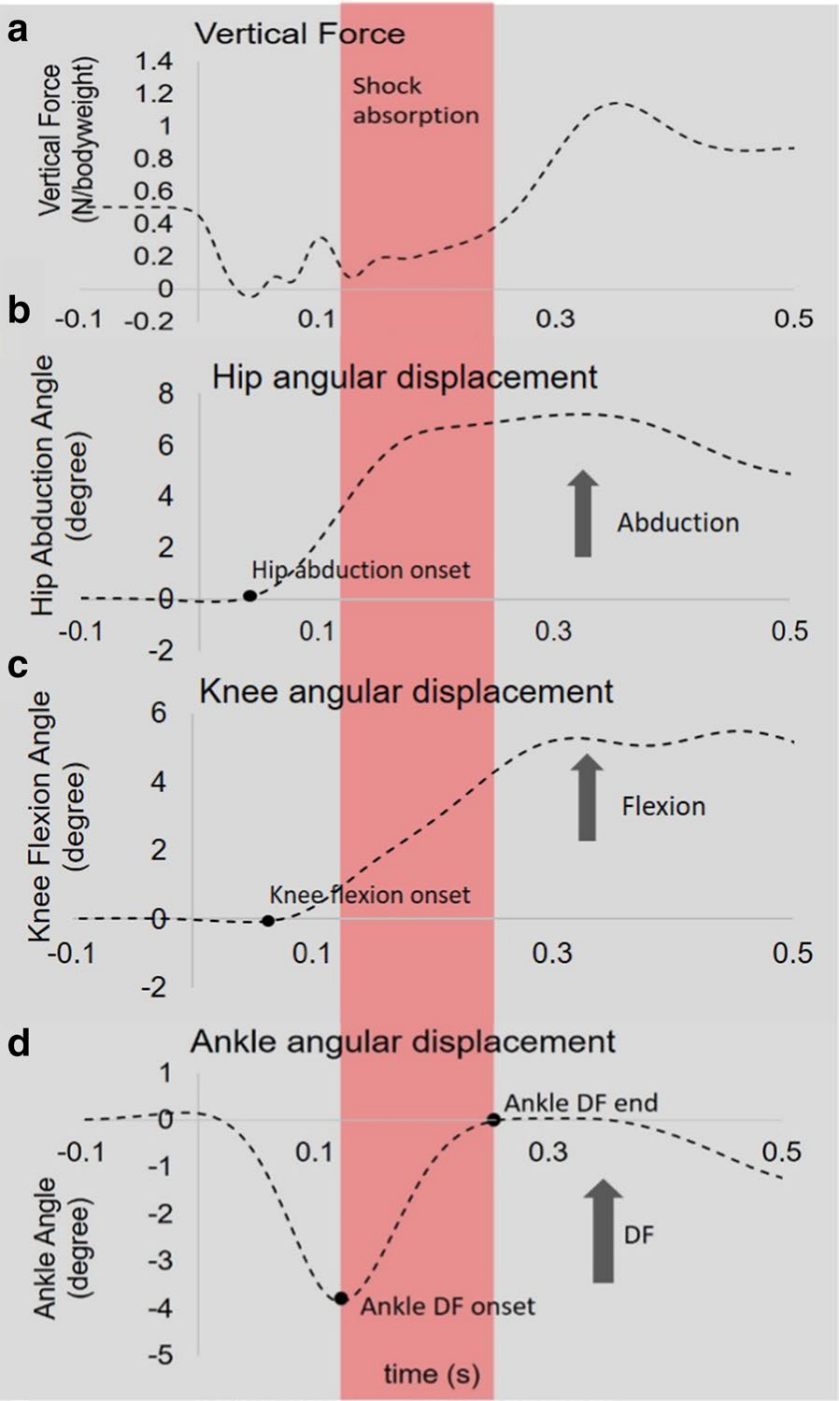
Representative data for vertical force (**a**), hip abduction (**b**), knee flexion (**c**) and ankle dorsiflexion (**d**) angular displacement for a control participant in a single trial. The area highlighted in red represents the shock absorption phase. Zero degree indicates upright standing position. DF: ankle dorsiflexion

Sagittal plane ankle and knee joint angular displacements and peak velocities were calculated during the shock absorption phase, which was defined as the time from maximal ankle plantarflexion to maximal dorsiflexion (see Fig. 2). Inter-joint timing for flexion onset was calculated as the difference between knee flexion onset time and ankle dorsiflexion onset time [36, 37]. Knee flexion onset timing was defined as the instant where the knee flexion angle reached the first minimum following perturbation. Similarly, ankle dorsiflexion timing onset was defined as the instant where ankle dorsiflexion angle reached the first minimum. Hip abduction angle was defined as the angle between the vector joining the right and left anterior and posterior superior iliac spine markers and the vector joining the knee and the greater trochanter markers projected to the frontal plane. Zero degree denotes upright standing position. Peak hip abduction angular displacement and peak angular velocity following perturbation were calculated. In addition, inter-joint timing between hip abduction and ankle dorsiflexion onset was calculated to study multi-planar coordination. Mean and standard deviation of the baseline hip angle prior to perturbation onset was calculated. Hip abduction onset timing was defined as the instant where the hip abduction angle exceeded 3 standard deviations over the baseline value following perturbation. Maximum trunk and body COM displacement in the vertical, anterior–posterior (AP), and medial–lateral (ML) directions were measured following perturbation. Body COM was estimated by calculating the average position between right and left anterior and posterior superior iliac spine [38]. The trunk segment was defined by using bilateral iliac crest and the acromion process markers [39]. The geometric center of the trunk was determined as the average position between the bilateral acromion and the anterior and posterior superior iliac spine markers. Paretic limb vertical ground reaction force (VGRF) was normalized to bodyweight and used to determine perturbation onset and the amplitude of weight-bearing. In addition, VGRF at the end of the shock absorption phase and at the maximal weight-bearing was measured.

Stabilization time of the COP velocity (COP_v_) was defined as the time elapsed from initial ground contact to the instant that COP_v_ settled within 3 standard deviations of its final stabilized value (as previously done in ground reaction forces stabilization analyses [40]). COP stabilization times in the medial–lateral (COP_v,M–L_) and the anterior–posterior (COP_v,A–P_) directions were calculated for the perturbed limb side. In addition, maximal stabilization time (COP_v,Max_) was determined by selecting the greater value between COP_v,M–L_ and COP_v,A–P_ stabilization time (i.e. the longer time).

Data averaged across trials from the paretic limb in the stroke group and the non-dominant limb in the control group were compared using 2-tailed t-tests. One-way analysis of variance (ANOVA) was used to compare the paretic, non-paretic and the control group non-dominant leg ST scores. We adjusted the analyses for multiple comparisons using the Benjamini–Hochberg procedure with a false discovery rate of 10% [41]. Pearson correlations were used to determine the relationships between COP_v,M–L_, COP_v,A–P,_ COP_v,Max_ stabilization times and CMSA score of the leg and foot subscale in individuals post-stroke. In addition, COP_v,M–L_, COP_v,A–P,_ COP_v,Max_ stabilization times and CMSA score of the leg and foot subscale were used in a bi-directional stepwise linear regression model to predict clinical ST and FSST scores for individuals post-stroke. The significance level was set at an α of 0.05. All statistics were determined using SPSS (version 25.0, SPSS, Inc).

## Results

All participants completed the Imposed Weight Transfer Assessment without wearing an AFO. Two participants with stroke wore an AFO during the ST and FSST. No falls occurred during the testing trials. No difference in joint angles prior to perturbation onset was identified between groups. In addition, no differences in ankle angle at dorsiflexion onset (stroke vs. control: 5.44 ± 4.5 vs. 5.59 ± 3.44, *p* = 0.92) and knee angle at flexion onset (stroke vs. control: 15.56 ± 7.53 vs. 11.47 ± 5.95, *p* = 0.11) were detected between groups. During the shock absorption phase, reduced ankle dorsiflexion, knee flexion, and hip abduction were observed in the stroke group compared to the control group (see Table 2). Peak angular velocity of ankle dorsiflexion, knee flexion, and hip abduction were decreased in individuals with stroke compared to controls. In addition, knee flexion onset timing relative to the onset of ankle dorsiflexion (inter-joint timing) was delayed in individuals with stroke compared to the control group. No difference in hip abduction onset timing relative to ankle dorsiflexion onset timing was detected between groups.

**Table 2:** Between-group comparisons in angular kinematics of the ankle, knee, and hip following perturbation

	Control	Stroke
**Angular displacement (degree)**		
Ankle_DF_	4.95(0.56)	3.39(0.48)*
Knee_FL_	7.27(0.96)	3.93(1.02)*
Hip_AB_	9.15(2.38)	6.62(2.62)*
**Angular velocity (degree/s)**		
Ankle_DF_	54.9(4.83)	42.35(3.39)*
Knee_FL_	85.97(8.11)	57.67(10.56)*
Hip_AB_	70.55(20.31)	54.83(17.84)*
**Inter-joint timing (ms)**		
Knee_FL,on_ – Ankle_DF,on_	− 35(9)	21(15)*
Hip_AB,on_ – Ankle_DF,on_	− 55(17)	− 75(4)

DF denotes dorsiflexion, FL denotes flexion, and AB denotes abduction. Negative inter-joint timing values indicate that the onset of knee flexion and hip abduction occurred before the ankle dorsiflexion

*Indicates p < 0.05

**Fig. 3:**
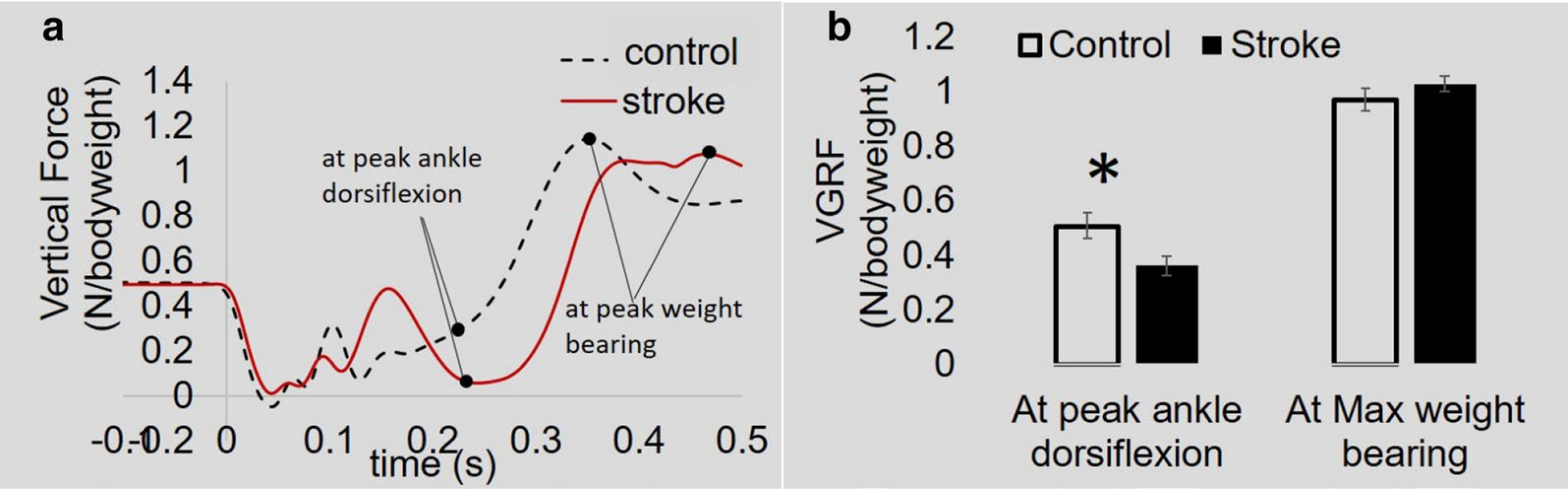
Vertical ground reaction force (VGRF) in stroke versus controls. **a** Representative vertical ground reaction force curves. During the shock absorption phase (~ 0.07–0.22 s), oscillation of vertical force was observed. Following the shock absorption phase, vertical force increased and eventually reached maximal weight-bearing. **b** Group comparisons of VGRF at peak ankle dorsiflexion and at maximal weight-bearing. *p < 0.05

Following landing, the vertical force oscillated during shock absorption and then increased to maximal weight-bearing (Fig. 3a). On average, VGRF at maximal weight-bearing was 0.93 ± 0.04 bodyweight in the control group and 1.03 ± 0.03 bodyweight in the stroke group (Fig. 3b). No differences in maximal weight-bearing were detected between groups. At peak ankle dorsiflexion (the end of the shock absorption phase), VGRF was reduced in the stroke group compared to controls (stroke: 0.36 ± 0.03 vs. control: 0.50 ± 0.05 bodyweight, *p* < 0.05).

Increases in peak vertical downward displacement of the trunk and the body COM were observed in the stroke group compared to controls (Table 3). No difference in trunk and body COM displacement was found in the A–P or M–L directions.

**Table 3:** Between-group comparisons of peak trunk and body center of mass (COM) displacement following perturbation

Peak displacement (mm)	Control	Stroke
**Vertical**		
Trunk (downward)	31.1 ± 7.0	38.7 ± 12.2*
Body COM (downward)	30.8 ± 7.1	38.3 ± 11.9*
**Anterior–posterior**		
Trunk (anterior)	9.3 ± 9.4	8.4 ± 6.9
Body COM (posterior)	28.1 ± 9.9	34.8 ± 13.6
**Medial–latera**l		
Trunk lateral	74.9 ± 18.4	76.4 ± 33.0
Body COM (lateral)	51.8 ± 17.5	59.0 ± 27.5

*Indicates p < 0.05

**Fig. 4:**
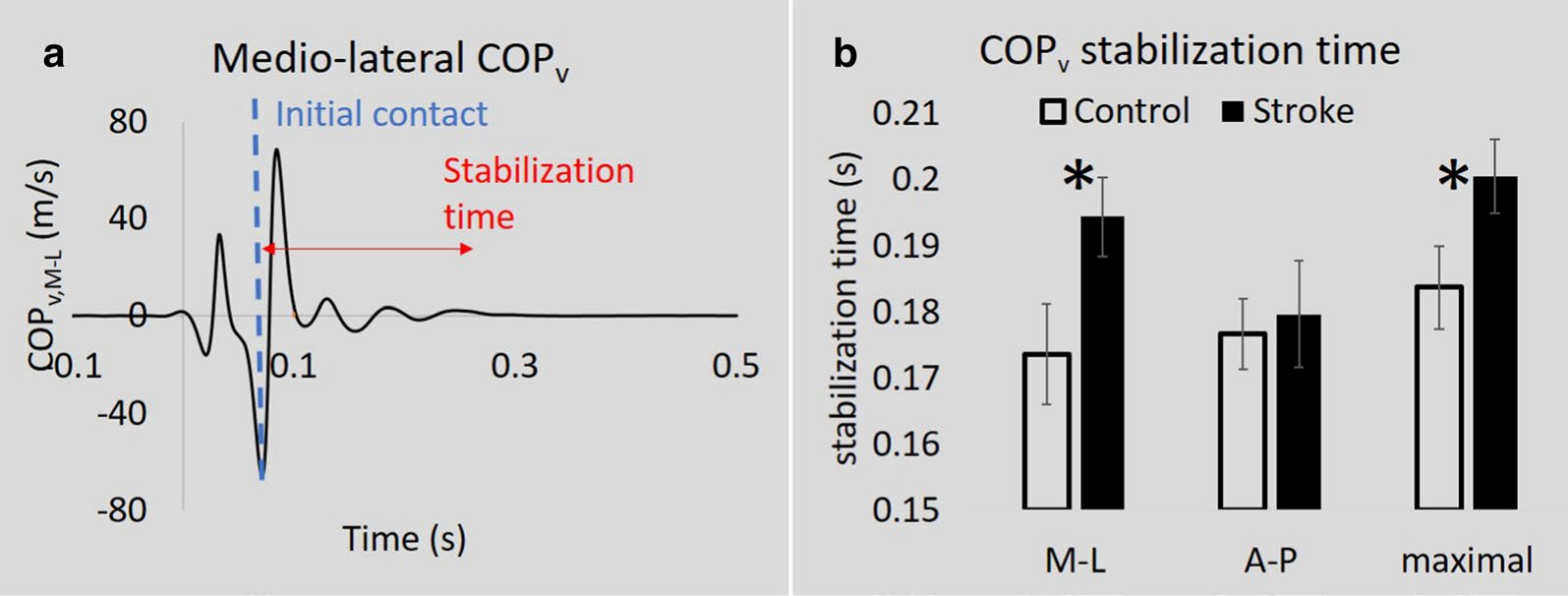
COP_v_ in stroke and controls. **a** Representative COP_v,M–L_ curve from a control participant. The dashed vertical line indicates the instant of initial contact. **b** COP_v_ stabilization time in the medial–lateral (M–L), anterior–posterior (A–P) directions, and the maximal stabilization time from the control versus stroke group. *p < 0.05

Participants with stroke showed prolonged COP_v,M–L_ and COP_v,Max_ stabilization time during weight transfer (COP_v,M–L_ stroke: 0.19 ± 0.01 s vs. control: 0.17 ± 0.01 s, *p* = 0.04, COP_v,Max_ stroke: 0.20 ± 0.01 s vs. control: 0.18 ± 0.01 s, *p* = 0.04, see Fig. 4). COP_v A–P_ stabilization time was not different between groups.

**Fig. 5:**
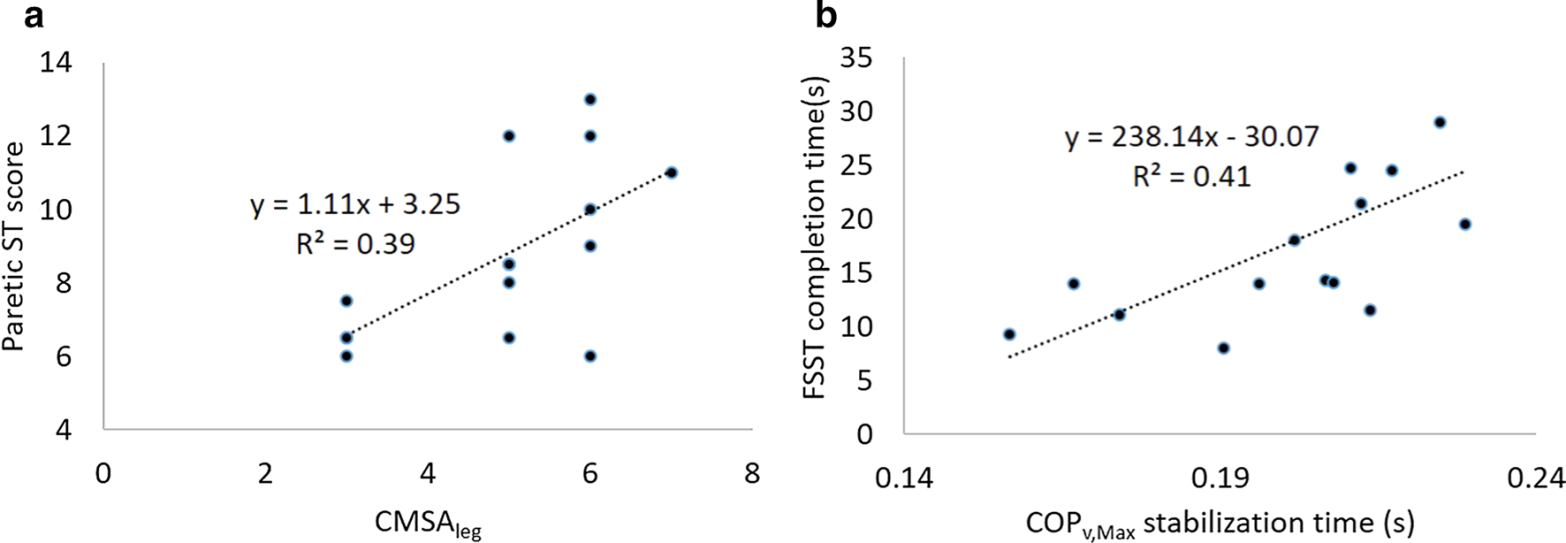
Predictors for clinical ST and FSST tests for all stroke participants. **a** CMSA of the leg explained 39% of the variance in paretic Step Test score. **b** COP_v,Max_ explained 41% of the variance in FSST completion time

During the ST, fewer steps were performed when stepping with the paretic limb (8.7 ± 0.6 steps) compared to the non-paretic limb (11.1 ± 0.6 steps, *p* < 0.01). Both paretic and non-paretic limb ST scores were lower compared to controls (16.3 ± 0.8 steps, *p* < 0.01). During the FSST, a longer time to complete the test was observed in the stroke group compared to the control group (stroke: 17.9 ± 2.0 s vs. control: 7.7 ± 0.4 s, *p* < 0.01). CMSA_foot_ was correlated with COP_v,A–P_ ( R^2^ = 0.28, p = 0.04). Stepwise linear regression performed for the stroke group revealed that CMSA_leg_ was the only predictor for the paretic ST score (Fig. 5a, R2 = 0.39, p = 0.01) and that COP_v,max_ predicted FSST (Fig. 5b, R2 = 0.41, p = 0.01). No predictor was identified for non-paretic ST score.

## Discussion

This study utilized a novel perturbation-based assessment device to characterize the biomechanical control of induced weight transfer in individuals post-stroke and age-matched healthy control participants. The findings confirmed our hypothesis that individuals post-stroke demonstrated reduced ankle dorsiflexion, knee flexion, and hip abduction during the shock absorption phase of weight transfer. In addition, the onset timing of knee flexion relative to ankle dorsiflexion was delayed for participants with stroke, indicating possible abnormalities in inter-joint coordination or joint stiffness during loading. Compared to controls, individuals with stroke took more time to stabilize the COP_v,M–L_ following ground contact. Prolonged paretic limb COP_v,Max_ stabilization time and lower CMSA score were associated with lower scores on clinical balance and mobility tests in stroke. These findings suggested that diminished lower limb joint flexion and hip abduction, inter-joint timing delays, and prolonged COP stabilization time likely reflected deficits in weight transfer and weight bearing ability following stroke**.**

Significantly greater passive resistance to dorsiflexion of the paretic ankle has been documented after stroke when perturbations were applied to the ankle joint during sitting [42]. Our results expanded this finding and demonstrated that when rapid weight transfer was induced, participants with stroke did not produce timely and sufficient joint flexion in the ankle and knee during the shock absorption phase. Potential factors that could contribute to reductions in joint flexion include muscle weakness and spasticity [43]. In particular, the hypertonicity of ankle plantarflexors has been well-documented in individuals with chronic stroke during passive ankle dorsiflexion in seated and supine positions [44, 45]. Spasticity could result in higher resistance to passive muscle stretch during dorsiflexion and lead to the reduced dorsiflexion displacement and angular velocity during limb loading observed in the present study. Based on the description of the CMSA foot subscale score, spasticity is present and marked in stages 2 and 3. By stage 5, spasticity has waned and is only evident with rapid movement or at the extremes of the range [46]. In our study more than half of the participants had a foot staged at 4 or less. In addition, lesion-induced alterations in intrinsic muscle properties [47–52] and/or architecture of the plantarflexors may cause an abnormal increase in passive ankle stiffness during dorsiflexion. Further research on the ankle and knee muscle properties and muscle activities during rapid weight transfer is warranted to determine the neuromuscular mechanisms underlying abnormal joint reactions during weight transfer in individuals post-stroke.

A coordinated spatio-temporal muscle synergy linking distal and proximal musculature is fundamental in providing an efficient postural response and in maintaining upright stability following external perturbations of standing. Following standing platform horizontal translations, a timing delay between the initiation of the proximal and distal muscle was 170% longer in post-stroke fallers compared to non-fallers [53]. This suggests that abnormalities in initiating muscle activity or intermuscular timing likely disrupts intralimb coupling and could contribute to falls in persons with stroke [53]. Our results supported the previous findings by demonstrating that knee joint flexion timing relative to the ankle was delayed in individuals post-stroke compared to controls. The inter-joint timing delays in flexion likely contributed to the disrupted weight transfer processes that may influence balance control. These findings highlight the importance of targeting problems of ankle-knee joint coordination during weight bearing activities for rehabilitation post-stroke.

Previous studies in landing have identified that trunk and lower extremity joint movements and neuromuscular activity are coordinated to effectively perform landing shock absorption [54, 55]. With reduced lower limb joint movements and disrupted inter-joint timing observed in participants with stroke, it is likely that more energy dissipation was required at the upper body. Thus, the increased trunk and body COM downward displacement observed in the stroke group may indicate that the upper body was destabilized partly due to the inefficient shock absorption from the lower limb. This observation may have important implications for gait performance. During the weight transfer phase of walking, the body COM is shifted forward and downward towards the leading limb following heel strike. An important function of the leading limb is to provide sufficient support and redirect the body COM forward and upward during this step-to-step transition. The mechanical work done during the step-to-step transition is a major determinant of the metabolic cost of walking [56]. If stroke-related deficits in ankle and knee impact absorption increase downward displacement of COM and mechanical work, a greater energy cost of walking would likely occur. However, although postural control principles of standing and gait are similar, the task performed in the present study was different from walking and, therefore, the abnormalities identified may or may not be directly applicable to continuous gait. In this regard, studies forcing weight transfer towards the paretic limb should also be conducted during steady state gait.

Our finding that individuals post-stoke showed abnormalities in ankle dorsiflexion during induced weight transfer is in agreement with previous gait studies that identified limited ankle dorsiflexion at initial contact and during stance in persons with stroke [43, 57]. In other continuous gait studies, premature ankle plantarflexor muscle excitation on the paretic side was observed and may prevent the knee from flexing further in response to early stance phase loading [58, 59]. During the weight acceptance phase of walking, contraction of the pretibial muscles restrains ankle plantarflexion and moves the tibia forward. This coordinated knee flexion and ankle dorsiflexion facilitates the weight transfer process to the leading limb and likely aides in maintaining higher instantaneous gait speed during loading response [60]. Without sufficient and timely joint flexion in the ankle and knee, greater body center of mass elevation is needed in order to advance the body over the heel fulcrum. A CMSA stage 4 and above indicates full active range of dorsiflexion and plantarflexion of the foot and full active range of knee flexion and extension. Based on the CMSA results, 7/15 and 11/15 participants had full active range of the foot and leg, respectively. Considering the importance of ankle and knee joints during weight bearing, it is likely that the reduced and slower knee and ankle joint flexion limited the ability to perform rapid weight transfer for individuals post-stroke during locomotion.

Previous studies suggest the importance of COP_v,M–L_ and amplitude as a tool for predicting fall risk [61] and balance control assessment [62]. In the present study, the perturbation imposed a rapid body COM displacement in the M–L direction challenging the individuals’ ability to control the COP_v,M–L_ and whole-body balance. Following ground contact, the COP_v,M–L_ was initially large and then decayed as balance became more stable. The prolonged time for COP_v,M–L_ to achieve a final steady-state level in individuals post-stroke reflects difficulties in balance control. For able-bodied individuals, the hip loading/unloading mechanism and control of ankle joint motion are the primary mechanisms for COP movement control [29]. After stroke, deficits in hip [14, 63, 64] and ankle [65] frontal plane movement control have been observed. This is consistent with our findings that hip abduction angular displacement and velocity were reduced and COP medial–lateral stabilization was delayed in participants with stroke. Thus, improving hip joint abduction/adduction, trunk, and ankle in/eversion control is likely important for improving COP control after a stroke. Findings from this research showed that in addition to abnormalities during vertical loading impact, difficulties in M–L balance control in individuals post-stroke also limit their weight transferability. These observations suggest that interventions aimed at improving weight transfer function in individuals post-stroke should consider both weight bearing and balance components during training.

The CMSA_foot_ was associated with COP_v,A–P_ stabilization time, likely because the majority of CMSA_foot_ assessment involved ankle dorsiflexion/plantarflexion movements and inversion/eversion was assessed in higher stages. Thus, motor recovery of the ankle is important for COP stabilization ability. In addition, CMSA_leg_ predicted paretic limb ST score. During paretic ST, paretic ankle and knee motion are key elements to move the paretic limb in and out of synergistic movements that were also assessed during the CMSA_leg_. Thus, lower extremity joint range of motion and the ability to move in and out synergistic patterns likely affected stepping performance. However, CMSA foot and leg subscales and COP_v_ measurements did not predict non-paretic ST score. During non-paretic ST, the paretic limb served as the stance limb. Unlike the CMSA and the induced weight transfer assessment, the flexion–extension coordination of the paretic stance limb has less influence on non-paretic ST scores. Rather, participants with stroke appeared to constantly extend the paretic limb during non-paretic ST. Thus, the induced weight transfer assessment and CMSA_leg_ better reflected the ability to coordinate flexion/extension movements rather than constant extension synergy alone. During the FSST, because multiple step directions were required, timely control of COM and COP movement in both A–P and M–L directions is essential for sustaining balance stability. Participants with stroke who required longer COP_v,max_ stabilization time during the imposed weight transfer assessment needed more time to complete FSST. These results confirmed that lower extremity joint coordination and COP_v_ stabilization in response to loading are key factors of functional weight transfer performance in individuals post-stroke. Given the relationship between the ST and FSST and fall risk [66], the imposed weight transfer assessment approach appeared to be useful in revealing possible mechanisms underlying deficits in balance and mobility following stroke.

Among the limitations of the present study was that the perturbation was designed to force vertical loading impact and challenge M–L balance control, however balance control in the A–P direction was not directly targeted. This may in part contribute to the COP_v,A–P_ stabilization time not being different in participants with stroke compared to controls. A more comprehensive setup to examine A–P, as well as M–L balance control, could be to provide the perturbation during a diagonal stance configuration as opposed to parallel foot placement. The diagonal orientation may also simulate the weight acceptance phase of gait. Another limitation of the present study is that joint torque/power and muscle activation patterns were not investigated. Thus, our results are consistent with but do not provide evidence to directly support abnormalities in neuromuscular control and intralimb coordination. Information regarding the effects of stroke on joint torque production during induced weight transfer is important to reveal the mechanism underlying abnormal joint reactions during weight bearing. Moreover, direct measurements of joint range of motion and muscle tone could provide important insight in addition to CMSA scores. In addition, because this study aimed to study the limb loading responses during individual’s natural stance, foot position was not standardized. Differences in initial stance width and shoe characteristics may influence the results. Finally, a limited number of participants with right hemiparesis and female participants with stroke were recruited for this study. Thus, further testing of a broader population of individuals post-stroke may enhance the generalizability of the results to these populations.

## Conclusions

The induced weight transfer paradigm demonstrated differences in the control of weight transfer in individuals post-stroke compare to controls. Decreased joint flexion of the ankle and knee, altered inter-joint timing, limitations in hip abduction and delayed COP stabilization may reflect difficulties in neuromuscular control during weight transfer following stroke. The induced weight transfer approach may potentially provide a useful means of training weight bearing and transfer problems in people with stroke.

## Data Availability

The datasets generated during and/or analyzed during the current study are available from the corresponding author on reasonable request.

## Abbreviations

COP: Center of pressure
COM: Center of mass
ST: Step Test
FSST: For-square Step Test
AFO: Ankle–foot orthosis
VGRF: Vertical ground reaction force
COP_v_: Center of pressure velocity
M–L: Medial–lateral
A–P: Anterior–posterior
COP_v,M–L_: Center of pressure velocity in the medial–lateral direction
COP_v,A–P_: Center of pressure velocity in the anterior–posterior direction
COP_v,max_: Maximal center of pressure velocity
